# Meta-analysis of the effects of oral and intravenous dexamethasone premedication in the prevention of paclitaxel-induced allergic reactions

**DOI:** 10.18632/oncotarget.13705

**Published:** 2016-11-29

**Authors:** Fu-chao Chen, Lin-hai Wang, Xiao-yu Zheng, Xiu-min Zhang, Jun Zhang, Lin-Jun Li

**Affiliations:** ^1^ Department of Pharmacy, Dongfeng Hospital, Hubei University of Medicine, Shiyan, Hubei, 442008, P.R. China; ^2^ Department of Pharmacy, Renmin Hospital, Hubei University of Medicine, Shiyan, Hubei, 442000, P.R. China; ^3^ International School of Software, Wuhan University, Wuhan, Hubei, 430079, P.R. China; ^4^ Department of Oncology, Renmin Hospital, Hubei University of Medicine, Shiyan, Hubei, 442000, P.R. China

**Keywords:** paclitaxel, dexamethasone, allergic reaction, meta-analysis

## Abstract

**Background:**

Dexamethasone premedication is required to prevent paclitaxel-related hypersensitivity reactions (HSRs). Oral dexamethasone (PO-D) has been considered the standard premedication regimen. However, whether intravenous dexamethasone (IV-D) is feasible for preventing paclitaxel-related HSRs is still unclear. We conducted a meta-analysis to compare these two regimens.

**Methods:**

We performed a systematic search in the PubMed, China National Knowledge Infrastructure, and Web of Science databases for relevant articles published before June 2016. Outcomes included HSRs and severe HSRs. Statistical analyses were performed using RevMan 5.2 software.

**Result:**

Six studies comprising 1347 patients were included in the meta-analysis. The PO-D premedication regimen showed a significantly decreased incidence of severe HSRs compared with the IV-D regimen with an OR of 0.53 (95% CI 0.28-0.99, p = 0.05). However, there was no difference in the overall paclitaxel-related HSR rates between the two premedication regimens (OR 0.76, 95% CI 0.55-1.06, p = 0.11). Subgroup analyses according to study type and country of origin showed similar statistical results between the two premedication regimens.

**Conclusion:**

Our meta-analysis showed that the PO-D premedication regimen is superior to the IV-D regimen in preventing paclitaxel-related HSRs. Additional randomized controlled trials are needed to confirm our findings.

## INTRODUCTION

Paclitaxel is a chemotherapeutic agent that acts as a microtubule stabilizer to inhibit cell division, which ultimately leads to cell death [1]. Paclitaxel has a wide antineoplastic spectrum and it is broadly used for the treatment of lung, ovarian, breast, head and neck, bladder, and other epithelial cancers [2]. The clinical use of paclitaxel is limited by its poor aqueous solubility. Therefore, it is usually formulated as an emulsion for intravenous (IV) delivery using 50% polyoxyethylated castor oil (Cremophor EL) and 50% ethanol as the solvent [3]. However, thisemulsion can lead to infusion-related hypersensitivity reactions (HSRs) [4]. The incidences of allergic and serious allergic reactions associated with paclitaxel administration are 0.7-7.7%. Some of these allergic reactions can be fatal [5, 6]. Therefore, effective premedications are usually administered to decrease the rate of allergic reactions and avoid the occurrence of severe allergic reactions due to paclitaxel treatment [7]. The standard premedication consists of dexamethasone, cimetidine, and diphenhydramine. Dexamethasone is orally administered 12 and 6 h before paclitaxel administration, whereas cimetidine and diphenhydramine are intravenously administered 30 min before paclitaxel administration [8]. Dexamethasone plays a pivotal role in preventing allergic reactions; however, it has also been associated with a number of adverse reactions. In addition, dexamethasone must be taken 12 and 6 h before chemotherapy. Therefore, forgetting to take one or both dexamethasone doses may be unsafe for patients. In view of this, experimental premedication with an IV dose of dexamethasone 30 min prior to paclitaxel administration has been implemented clinically. However, the results of the systematic reviews on the therapeutic effects of these different regimens are unclear [9].

In this report, we analysed the effects of two common administration routes of dexamethasone premedication on the prevention of paclitaxel-induced HSRs. We also evaluated the effectiveness of each route to provide an evidence-based reference for physicians.

## RESULTS

### Selected studies

**Figure 1:**
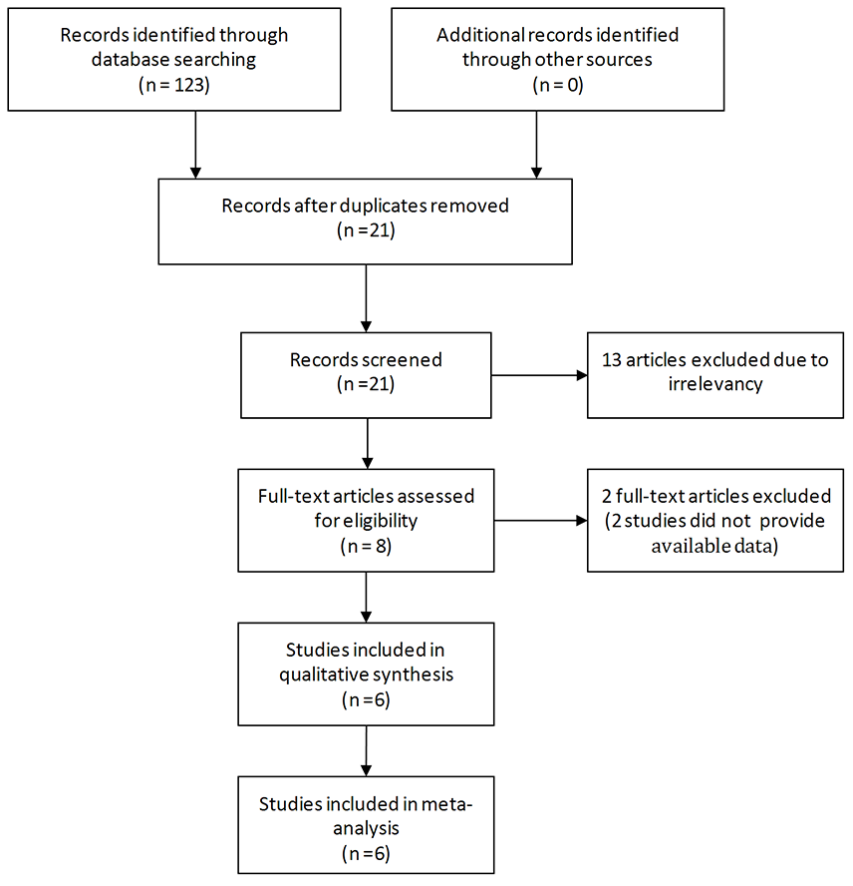
Flow diagram of the study selection process

Of the studies initially identified, we excluded reports that did not meet the inclusion criteria after first screening the study titles and abstracts. Six studies [9–14] were ultimately included in the meta-analysis. Figure 1 illustrates how the six selected studies were obtained from the literature search.

### Study characteristics and quality assessment

The six selected studies were published between 1996 and 2013, and including two in Canada [9], two in China [12, 13], one in Sweden [11] and one in Italy [10]. The sample sizes of the included studies ranged from 137 to 483 patients, comprising a total of 1347 included patients. The main characteristics of the studies are listed in Table 1. Five of the six inclusion studies were retrospective studies, with the sixth study published by P Rosenberg classified as a randomized controlled trial [11], but none of these studies described the random allocation method in detail or specified if there was blinding or allocation concealment. In addition, three of the studies only recorded the incidences of allergic (Common Terminology Criteria for Adverse Events version 3.0, grades 1 to 2) and severe allergic reactions (Common Terminology Criteria for Adverse Events version 3.0, grade 3) and were therefore missing a detailed description of the final indicator [14]. Table 1 shows the Newcastle–Ottawa scores and the modified Jadad scale for the quality assessment of the non-randomized studies and randomized studies.

**Table 1:** Summary of the characteristics of the studies included in the meta-analysis

Study	First author	Publication year	PO-D			IV-D			Quality score
			SAA	AA	Total	SAA	AA	Total	
1	Gennari A	1996	1	7	90	1	3	47	6
2	Kwon JS	2002	1	8	107	8	19	110	8
3	Hua XM	2004	8	24	212	12	34	271	5
4	P Rosenberg	2002	5	29	106	5	29	99	4
5	Chen Y	2013	0	3	77	0	1	80	5
6	Sean M	2013	0	5	93	3	8	55	7

**Abbreviations: AA, allergic reaction; IV-D, intravenous dexamethasone; PO-D, oral dexamethasone; SAA, serious allergic reaction**

### Main results of meta-analysis

#### Comparison of the HSR rates from the PO-D and IV-D treatments

**Figure 2:**
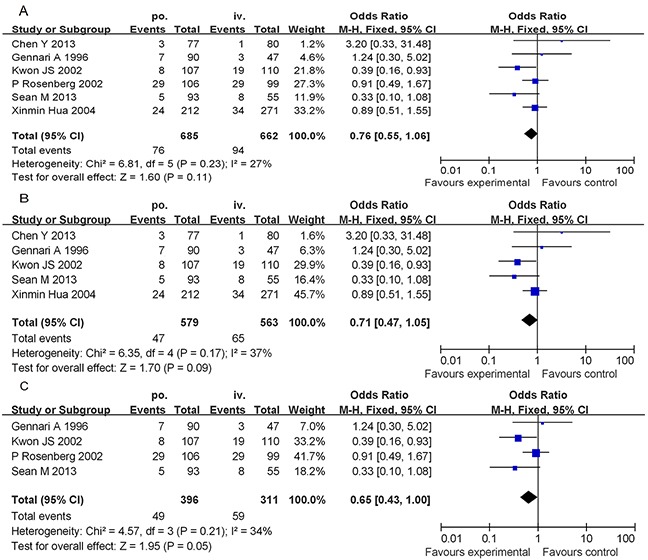
Forest plots of the meta-analysis of the allergic reactions in patients administered PO-D regimen compared with reactions in patients administered the IV-D regimen Notes: **A.** overall studies; **B.** subgroup analyses (excluding the randomized controlled trial); **C.** subgroup analyses (excluding the People's Republic of China). Abbreviations: IV-D, intravenous dexamethasone; CI, confidence interval; PO-D, oral dexamethasone.

The six studies included 1347 patients; of these, 94 (14.20%) patients in the IV-D group and 76 (11.09%) patients in the PO-D group experienced an HSR to paclitaxel (Figure 2A). We used a fixed effects model to analyse the results owing to the moderate inconsistency between the two groups of study results (χ^2^ = 6.81, P = 0.23, I^2^ = 27%). The combined OR (95% CI) was 0.76 (0.55-1.06), which indicates no statistically significant association between the PO-D and IV-D treatments. In a further investigation, subgroup analyses were performed. When stratified by “study type,” the “retrospective studies” group yielded an OR of 0.71, and the 95% CI was 0.47–1.05 (Figure 2B). In the subgroup analysis by “country,” the “non-Chinese studies” group yielded an OR of 0.65 with a 95% CI of 0.43–1.00 (Figure 2C).

#### Comparison of the severe HSR rates from the PO-D and IV-D treatments

**Figure 3:**
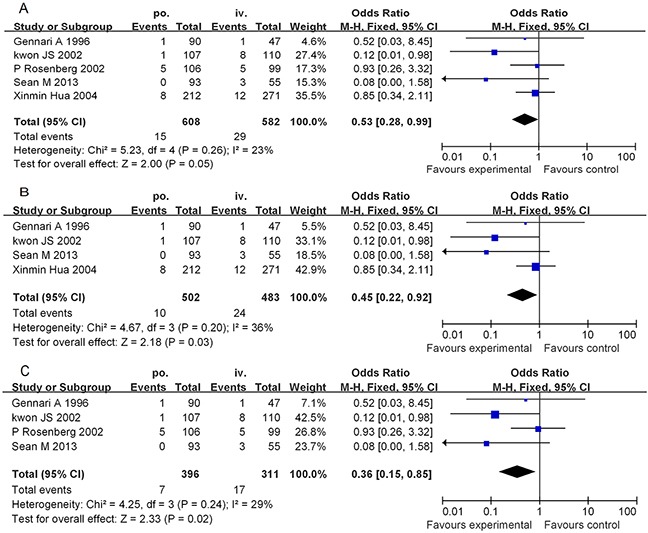
Forest plots of the meta-analysis of the severe allergic reactions in patients administered the PO-D regimen compared with in patients administered the IV-D regimen Notes: **A.** overall studies; **B.** subgroup analyses (excluding the randomized controlled trial); **C.** subgroup analyses (excluding the People's Republic of China). Abbreviations: IV-D, intravenous dexamethasone; CI, confidence interval; PO-D, oral dexamethasone.

The rates of severe HSRs from pretreatment with PO-D versus pretreatment with IV-D were 2.47% (15/608) versus 4.98% (29/582), respectively, (Figure 3A). We used a fixed effects model to analyse the results owing to the moderate inconsistency between the two groups of study results (χ^2^ = 5.23, P = 0.26, I^2^ = 23%). The combined OR (95% CI) was 0.53 (0.28-0.99), which indicates a statistically significant association between the PO-D and IV-D treatments. In the subgroup analysis by study type and country, the combined ORs were 0.45 (95% CI: 0.22–0.92, P = 0.03) for the retrospective studies (Figure 3B) and 0.36 (95% CI: 0.15–0.85, P = 0.02) for the non-Chinese studies (Figure 3C). According to Figure 3, the number of severe HSRs from PO-D premedication accounted for 36-53% of those from IV-D premedication. This indicates that the incidence of severe HSRs due to PO-D premedication is significantly lower than that from premedication with IV-D in preventing paclitaxel-induced HSRs.

### Publication bias

**Figure 4:**
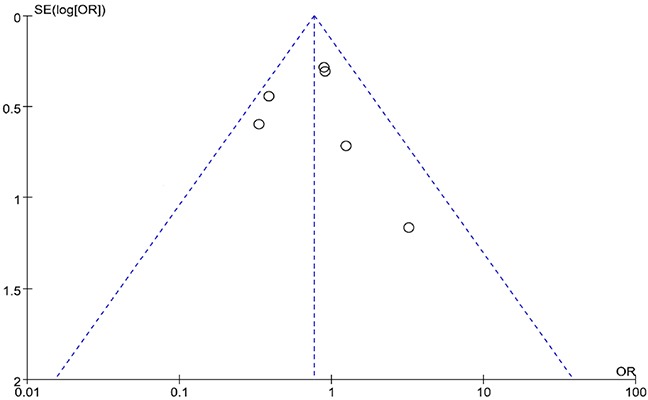
Funnel plots of the six selected studies

Regarding publication bias, there was an obvious asymmetry of the funnel plot (Figure 4), which suggests that there was some level of publication bias. However, because the number of included studies was only six, the funnel plots may not be significant. Egger's test revealed that publication bias was not significant in both overall HSR (P = 0.073) and severe HSR (P = 0.195) analysis.

## DISCUSSION

Paclitaxel is a natural diterpenoid extracted from the stem bark of *Taxus brevifolia* and has been a research focus for decades due to its unique therapeutic effects and excellent anticancer activities [1–3]. By promoting tubulin assembly and stabilizing microtubules, paclitaxel can inhibit mitosis and result in the apoptosis of tumour cells [15–16]. Due to paclitaxel's lack of aqueous solubility, the drug is usually solubilized in polyoxyethylated castor oil (Cremophor EL) [17]; however, this can cause HSRs because Cremophor EL induces the release of histamine. Paclitaxel-induced HSRs are consistent with type I HSRs, which are caused by immunoglobulin E-mediated release of histamine and leukotrienes from mast cells [18–21]. It has been reported that severe HSRs are characterized by chest pain, dyspnoea, bronchospasm, urticaria, abdominal cramping, and hypertension [22], all of which can be life threatening at times. In addition, if severe HSRs occur, paclitaxel treatment is discontinued, which is disadvantageous for patients; thus, prophylactic treatments have been used. Dexamethasone is a long-acting synthetic glucocorticoid with a biological half-life of approximately 48 h. Dexamethasone exerts its effects by diminishing cytokine production by tumours [23], increasing the expression level of tumour necrosis factor [24], and decreasing the expression levels of interleukin 1 beta [25] and vascular endothelial growth factor [26, 27]. Dexamethasone can alleviate myelosuppression and enhance the antitumour effects based on this activity. Thus, it is an essential drug that is widely administered with chemotherapeutic agents to alleviate the toxic side effects of chemotherapy [27–29].

For patients treated with paclitaxel, the US Food and Drug Administration has approved premedication with oral dexamethasone (20 mg, administered 12 and 6 h before paclitaxel administration), oral or IV diphenhydramine (25-50 mg), and an H_2_ antagonist. Diphenhydramine and the H_2_ antagonist are commonly administered 30-60 min prior to paclitaxel infusion. However, it has been reported that patients are noncompliant with and inconvenienced by the abovementioned oral dosing schedules. In addition, overuse of the premedication regimen can critically desensitize cells against chemotherapy and induce HSRs [30]. Some studies [16, 31] have shown that pretreatment with dexamethasone can inhibit the therapeutic efficacy of paclitaxel. The inhibitory effect of a 20 mg/kg dose of paclitaxel on the growth of breast and ovarian xenograft tumours was found to be reduced by approximately 20-25% when animals were pretreated with 1 mg/kg dexamethasone [16]. This observation prompted investigations into developing more convenient but equally safe alternative dexamethasone regimens. Premedication with IV-D (administered 30 min before paclitaxel infusion) precludes the oral intake of dexamethasone on the night before and on the morning of paclitaxel treatment. This can improve patient compliance and, to a certain degree, the patient's quality of life.

The data obtained from the meta-analysis indicates that the incidence of HSRs and severe HSRs due to PO-D premedication was lower than that due to IV-D premedication. Therefore, we suggest that the PO-D regimen should be the preferred regimen to minimize paclitaxel infusion-related HSRs. However, prophylaxis with IV-D (10-20 mg, administered 30 min before paclitaxel administration) may be necessary in the following conditions: (i) if the patient is noncompliant, (ii) if the patient forgot to take either or both oral doses of the premedication, and (iii) if higher doses of PO-D are required, which can induce some adverse reactions. The latter is especially important for patients with diabetes or osteoporosis. Paclitaxel-related HSRs occurring after IV-D administration should be immediately and appropriately managed by using the following techniques: (i) stopping the paclitaxel infusion but continuing to administer IV normal saline at 200 cc/h to maintain and stabilize blood pressure; (ii) administering oxygen at 2-4 L/min via nasal cannula to maintain oxygenation; (iii) administering an IV push of 125 mg methylprednisolone to counteract respiratory distress; (iv) administering an IV push of 50 mg diphenhydramine to counteract respiratory distress and inflammation; (v) continuously monitoring blood pressure, pulse, and oxygen saturation; and (vi) immediately notifying the physician for further orders and initiating a code if either airway patency is not maintained or cardiopulmonary arrest occurs [32]. Beyond these recovery methods, we should reevaluate additional research that is available regarding paclitaxel infusion-associated HSRs because the solvent and dosage formulations are increasingly changing.

O’Cathail SM et al [9] reported on the management of paclitaxel-induced HSRs. The authors compared the incidence of HSRs resulting from administration of the conventional prophylactic regimen of two oral doses of corticosteroids with the incidence from administering a single IV dose of a corticosteroid, which was similar to our meta-analysis. In that study, only two previous reports, both of which were English articles, were reviewed in addition to their own experience on the subject. The authors observed a statistically significant difference between the frequencies of HSRs from the two different regimens. However, only a few severe HSRs were observed in the studies, and a meta-analysis of severe HSRs was not performed. In this study, we analysed six independent studies, including two Chinese articles, obtained from searches of English and Chinese databases.

The data obtained from our meta-analysis indicate that premedication with either PO-D or IV-D can prevent HSRs and severe HSRs due to paclitaxel administration. However, compared with the standard regimen of administering PO-D at 12 and 6 h before paclitaxel infusion, administering IV-D 30 min before paclitaxel infusion is associated with a higher rate of HSRs. The differences in the rates of severe HSRs between the two administration routes were found to be statistically significant (P = 0.05). In the subgroup analyses according to study type and country, similar outcomes were observed between the two dexamethasone premedication protocols. This suggests that PO-D protocols are the better premedication option.

Our meta-analysis had several limitations. First, because only six studies met our inclusion criteria, publication bias could not be completely excluded based on the low power of the funnel plot asymmetry and Egger's test. Second, the number of included patients was small, and the lower quality of the non-randomized and randomized studies could make the conclusion less convincing. Third, one study [12] used a different study protocol as well as different doses of dexamethasone, which could have caused significant heterogeneity in the data; however, the heterogeneity from our data analysis was not significant. Therefore, large-scale and well-designed randomized controlled studies are needed to verify these results.

In summary, our meta-analysis shows there was no significant difference between PO-D and IV-D administration in preventing HSRs. The PO-D protocol could significantly decrease the risk of severe HSRs induced by paclitaxel compared to IV-D administration. More randomized controlled trials with larger sample sizes should be conducted to confirm these findings.

## MATERIALS AND METHODS

### Search strategy

We performed a systematic literature search in PubMed, China National Knowledge Infrastructure, and Web of Science databases using the following keywords: “paclitaxel,” “dexamethasone,” “allergic reactions,” and “hypersensitivity reactions.” We then assessed the reference lists of the selected articles for studies that compared the effects of premedication with oral and IV dexamethasone on preventing paclitaxel-induced allergic reactions. We selected randomized and non-randomized trials provided they were well designed.

### Inclusion criteria

Published studies were selected for analysis based on the following criteria: (i) use of human subjects, (ii) study design (controlled clinical study comparing the effects of two administration routes of dexamethasone on preventing paclitaxel-induced allergic reactions), and (iii) dose of dexamethasone. We selected studies in which oral dexamethasone (PO-D; 10-20 mg, given 12 and 6 h before paclitaxel administration) and IV dexamethasone (IV-D; 10-20 mg, given 30 min before paclitaxel administration) were assigned to the control and experimental groups, respectively.

### Exclusion criteria

The exclusion criteria were as follows: (i) original studies that did not compare the effects of two administration routes of dexamethasone on preventing paclitaxel-induced allergic reactions and (ii) original studies that did not meet criterion (i) but in which different doses of dexamethasone were administered to the experimental and control groups.

### Study selection and data extraction

Two reviewers independently screened the titles and abstracts of all identified articles and excluded studies that clearly did not meet the criteria. A second screening was based on a full text review. Information collected from these publications included the following: first author's name, publication year, study design, sample size, and quality evaluation. Next, we extracted all data using a standardized data extraction form. Differences were resolved through discussions with a third independent expert.

### Quality evaluation

To determine the validity of the selected studies, a modified Jadad scale was used to assess the quality of the included randomized studies [33]. High quality studies have scores of 4-8, whereas low quality studies have scores of 0-3. For non-randomized studies, the Newcastle-Ottawa Quality Assessment Scale was used [34]. Each study was graded as either low quality (0-5) or high quality (6-9).

### Statistical analysis

All statistical meta-analyses were conducted using RevMan 5.2 software, which was provided by Cochrane (London, UK). Heterogeneity among the studies was evaluated using the X^2^ test. An alpha level of 5% was used to designate statistical significance. We used a fixed effects model to analyse results when there was no significant heterogeneity (P > 0.05, I^2^ ≤ 50%). On the other hand, a random effects model was used when there was significant heterogeneity (P < 0.05, I^2^ ≤ 50%) but no clinical differences. Pooled odds ratios (ORs) and 95% confidence intervals (CIs) were calculated for the categorical outcomes using the Mantel-Haenszel fixed effects model because there was no evidence of significant heterogeneity for the outcomes.
